# The essential enterprise: the critical role of accreditation in the 21st century

**DOI:** 10.1186/s12909-020-02120-6

**Published:** 2020-09-28

**Authors:** Jason R. Frank, Sarah Taber, Marta van Zanten, Fedde Scheele, Danielle Blouin, on behalf of the International Health Professions Accreditation Outcomes Consortium

**Affiliations:** 1 Office of Specialty Education, Royal College of Physicians and Surgeons of Canada, Ottawa, Canada; 2 Department of Emergency Medicine, University of Ottawa, Ottawa, Canada; 3 Foundation for Advancement of International Medical Education and Research, Philadelphia, PA USA; 4 OLVG Teaching Hospital, Amsterdam, The Netherlands; 5 VU Medical Center, School of Medical Sciences, Amsterdam, The Netherlands; 6 Athena Institute for Transdisciplinary Research, Amsterdam, The Netherlands; 7 Department of Emergency Medicine, Queen’s University, Kingston, Canada

Health professions education (HPE) is undergoing rapid change to a competency-based world, and accreditation change is part of that story [1–4]. Despite over 100 years of scientific, instructional, and biomedical innovation, health professions education continues to face criticism. Deficits and variations in graduate competence, patient harm, and preparedness for modern health care are considered major challenges for current designs for HPE [5, 6]. Can accreditation help to address these issues?

Accreditation is commonly viewed as an essential component of an effective health professions education (HPE) system, valued both as a lever for quality assurance as well as for continuous quality improvement. However, for such an essential enterprise, the body of literature on HPE accreditation is small. Accreditation systems exist worldwide in a wide variety of forms. Do we all agree on what we mean by “accreditation”? What are the essential components of an accreditation system? What works best for a given context? What are the emerging issues in contemporary education? What are best and “next” practices? We have only the work of a few pioneering scholars to inform these questions, and no global consensus on which to advance our thinking.

Enter an accreditation community of practice, the International Health Professions Accreditation Outcomes Consortium (IHPAOC). We founded this organization in 2012 to advance the practice of HPE accreditation.

To date, this group has organized two world summits on HPE accreditation, one in 2013 in conjunction with the International Conference on Residency Education (ICRE) in Calgary, Canada, and the second in 2018 in conjunction with the Association for Medical Education in Europe (AMEE) conference in Basel, Switzerland. Both summits used an iterative group process to identify themes related to the current state of and future directions for HPE accreditation.

At its first world summit in 2013, IHPAOC members from around the world, including Canada, the United States, Australia, Europe and China, began a collaborative process of discussion and consensus-building to identify priority topics and build a research agenda. Working groups were formed to further elaborate on the identified topics and each group produced a paper. BMC Medical Education was chosen as vehicle to widely disseminate the IHPOAC findings in an open access format. This is the resulting accreditation paper series.

Each paper makes a primary contribution to the literature on accreditation. The first paper in this series by Frank et al. presents a new definition of accreditation, examines its purpose and return on investment, and defines 10 fundamental and recurring elements of accreditation systems commonly found in HPE. The second paper by Taber et al. provides a framework for operational design decisions for accreditation systems. The third paper, by Bandiera et al., explores the need for accreditation systems to shift their focus from processes to outcomes. The fourth paper in the series by Akdemir et al. examines the role of continuous quality improvement and develops a core values framework for accreditation. The last paper, written by Philibert et al., describes four priorities to enhance the societal responsiveness of HPE, and identifies approaches at the system, institution, program and individual levels using accreditation as a lever for change.

Many thanks to all the participants in the IHPOAC community for all their contributions. It is our hope that this supplement provides a valuable contribution to the accreditation enterprise. Ultimately, the consortium’s goal is to continue to build a body of scholarship connecting good practice in accreditation and impact on health systems and health outcomes.

## Data Availability

Not applicable.

## Abbreviations

AMEE: Association for Medical Education in Europe
HPE: Health professions education
ICRE: International Conference on Residency Education
IHPAOC: International Health Professions Accreditation Outcomes Consortium
